# Somatically expressed germ-granule components, PGL-1 and PGL-3, repress programmed cell death in *C. elegans*

**DOI:** 10.1038/srep33884

**Published:** 2016-09-21

**Authors:** Mohammad Al-Amin, Hyemin Min, Yhong-Hee Shim, Ichiro Kawasaki

**Affiliations:** 1 Department of Bioscience and Biotechnology, Konkuk University, Seoul, Republic of Korea

## Abstract

We previously reported that germline apoptosis in *C. elegans* increased by loss of PGL-1 and PGL-3, members of a family of constitutive germ-granule components, from germ cells in adult hermaphrodite gonads. In this study, we found that somatic apoptosis was reduced in synthetic multivulva class B (synMuv B) mutants due to ectopic expression of PGL-1 and PGL-3 in the soma. In synMuv B-mutant somatic cells, CED-4 expression level was reduced due to ectopic expression of PGL-1. Furthermore, in contrast to wild type, somatic apoptosis in synMuv B mutants increased following DNA damage in a SIR-2.1-dependent manner. Intriguingly, somatic apoptosis was repressed not only in synMuv B mutants but also by ectopically expressing *pgl-1* and/or *pgl-3* transgenes in wild-type somatic cells. Our study demonstrates that germ-granule components, PGL-1 and PGL-3, can serve as negative regulators of apoptosis not only in the germline but also in the soma in *C. elegans*.

Germ cells are distinct from somatic cells in their immortality, totipotency, and ability to undergo meiosis. Germ cells of various organisms contain distinctive cytoplasmic organelles called germ granules, which are made of RNAs and RNA-binding proteins^1^,^2^. Because of their presence in diverse organisms, germ granules are considered to play pivotal roles in germline development possibly through conferring special characteristics on germ cells. P granules, the *C. elegans* germ granules^3^,^4^, are required for proper postembryonic germline development^5^,^6^,^7^. We previously reported that germline apoptosis increased by loss of PGL-1 and PGL-3, members of a family of constitutive P-granule components^5^,^6^, from germ cells in adult hermaphrodite gonads^8^.

In synthetic multivulva class B (synMuv B) mutants, somatic cells acquire germline-like characteristics and express germline-specific proteins including PGL-1 and PGL-3 especially at elevated temperatures^9^,^10^,^11^. It was previously reported that a number of synMuv B mutants showed decreased somatic cell death by an unknown mechanism^12^. Since we found that PGL-1 and PGL-3 repress apoptosis in the germline^8^, we hypothesized that ectopic expression of PGL-1 and PGL-3 in synMuv B-mutant somatic cells might antagonize programmed cell death in the soma. Here we show that somatic apoptosis decreases by ectopic expression of PGL-1 and PGL-3 in somatic cells, indicating that PGL-1 and PGL-3 can serve as negative regulators of apoptosis not only in the germline but also in the soma in *C. elegans*. Our finding brings up a fascinating possibility that expression of a germline program in somatic cells can provide the soma with partial germline immortality at a cellular level.

## Results and Discussion

### Somatic apoptosis was reduced in *hpl-2* mutants due to ectopic expression of PGL-1 in somatic cells

**Figure 1:**
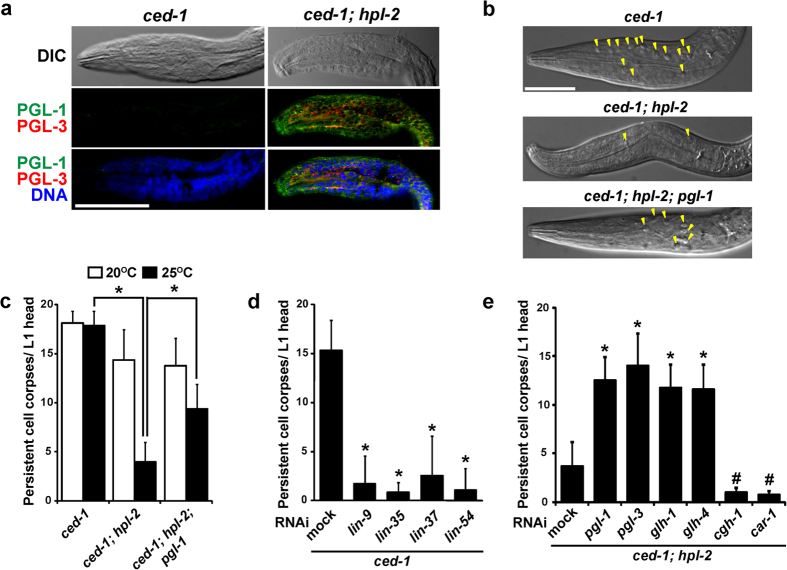
Somatic apoptosis was reduced in synMuv B mutants due to ectopic expression of P-granule component genes in somatic cells. (**a**) Ectopic expression of PGL-1 and PGL-3, members of a family of germline-specific P-granule components, in the L1 larval head of *ced-1(e1735); hpl-2(tm1489)* double, but not *ced-1(e1735)* single, mutants grown at 25 °C. Differential interference contrast (DIC) and immunofluorescence images double-immunostained with anti-PGL-1 (green) and anti-PGL-3 (red) antibodies along with TO-PRO-3 DNA staining (blue). Scale bar: 20 μm. (**b**) L1 larval heads of *ced-1(e1735)* single, *ced-1(e1735); hpl-2(tm1489)* double, and *ced-1(e1735); hpl-2(tm1489); pgl-1(ct131)* triple mutants displayed different numbers of persistent cell corpses at 25 °C. Some of the cell corpses are marked with yellow arrowheads. Scale bar: 20 μm. (**c**) Mean ± s.d. numbers of persistent cell corpses per L1 head (n > 50) in *ced-1(e1735)* single, *ced-1(e1735); hpl-2(tm1489)* double, and *ced-1(e1735); hpl-2(tm1489); pgl-1(ct131)* triple mutants grown at either 20 °C (white bars) or 25 °C (black bars). *P < 0.05. (**d**) Mean ± s.d. numbers of persistent cell corpses per L1 head at 25 °C (n > 50) in *ced-1(tm2420)* mutants after RNAi depletion of *lin-9*, *lin-35*, *lin-37*, and *lin-54* gene, respectively, with mock RNAi control. *P < 0.05 compared with mock RNAi. (**e**) Mean ± s.d. numbers of persistent cell corpses per L1 head at 25 °C (n > 50) in *ced-1(tm2420); hpl-2(tm1489)* double mutants after RNAi depletion of *pgl-1*, *pgl-3*, *glh-1*, *glh-4*, *cgh-1*, and *car-1* gene, respectively, with mock RNAi control. *^,#^*P* < 0.05 compared with mock RNAi.

To measure somatic apoptosis, we scored the number of persistent cell corpses in the heads of newly hatched L1 larvae using Nomarski differential interference contrast (DIC) microscopy in *ced-1* mutant background, in which engulfment of cell corpses is blocked, hence a sensitive condition to score cell corpses is provided^13^,^14^. Among synMuv B-class mutants, we mainly examined *hpl-2* mutants^15^. First, we confirmed that both PGL-1 and PGL-3 were ectopically expressed in somatic tissues, including the head part, in newly hatched L1 larvae of *ced-1; hpl-2* double, but not *ced-1* single, mutants at an elevated temperature, 25 °C (Fig. 1a). We found that the number of cell corpses in the L1 head was significantly reduced in *ced-1; hpl-2* double mutants compared to *ced-1* single mutants at 25 °C but not at 20 °C (Fig. 1b,c). We consider that the reason why the number of cell corpses was more decreased at 25 °C than at 20 °C in *ced-1; hpl-2* double mutants was because there were more PGL-1 and PGL-3 proteins at 25 °C than at 20 °C in *hpl-2* mutant somatic cells, as previously described^9^. Furthermore, this reduced somatic apoptosis in *ced-1; hpl-2* double mutants was significantly recovered in *ced-1; hpl-2; pgl-1* triple mutants at 25 °C (Fig. 1b,c). These results suggest that somatic apoptosis is reduced in *hpl-2* mutants due to ectopic expression of PGL-1 in somatic cells.

### Somatic apoptosis was reduced by RNAi depletion of synMuv B-class genes, and reduced somatic apoptosis in *hpl-2* mutants was recovered by RNAi depletion of a subset of P-granule component genes

To examine whether depletion of other synMuv B-class genes also causes a reduction in somatic apoptosis, we scored the number of cell corpses in the L1 heads of *ced-1* mutants after RNAi depletion of several synMuv B-class genes at 25 °C (Fig. 1d). We found that the level of somatic apoptosis was significantly reduced, as in *ced-1; hpl-2* double mutants, after RNAi depletion of *lin-9*, *lin-35*, *lin-37*, and *lin-54* (Fig. 1d), all of which are synMuv B-class genes, and depletion of them was previously shown to cause ectopic expression of PGL-1 and PGL-3 in the soma^9^. These results indicate that reduction of somatic apoptosis is a common characteristic among synMuv B-class mutants. We also examined whether depletion of other P-granule component genes recovers the reduced somatic apoptosis in *ced-1; hpl-2* double mutants, as did *pgl-1* mutation (Fig. 1b,c,e). We found that the reduced somatic apoptosis in *ced-1; hpl-2* double mutants was significantly recovered after RNAi depletion of *pgl-1*, *pgl-3*, *glh-1*^16^, and *glh-4*^7^, but not after RNAi depletion of *cgh-1*^17^ and *car-1*^18^ (Fig. 1e). These results indicate that reduced somatic apoptosis in synMuv B-class mutants is recovered by depletion of a subset of P-granule component genes. We confirmed that our RNAi treatments indeed diminished the expression or activity of target genes through the observation that all the RNAi treatments successfully phenocopied previously reported loss-of-function mutant phenotypes of these genes (Figs S1 and S3c).

### CED-4 expression was reduced in *hpl-2*-mutant somatic cells due to ectopic expression of PGL-1

**Figure 2:**
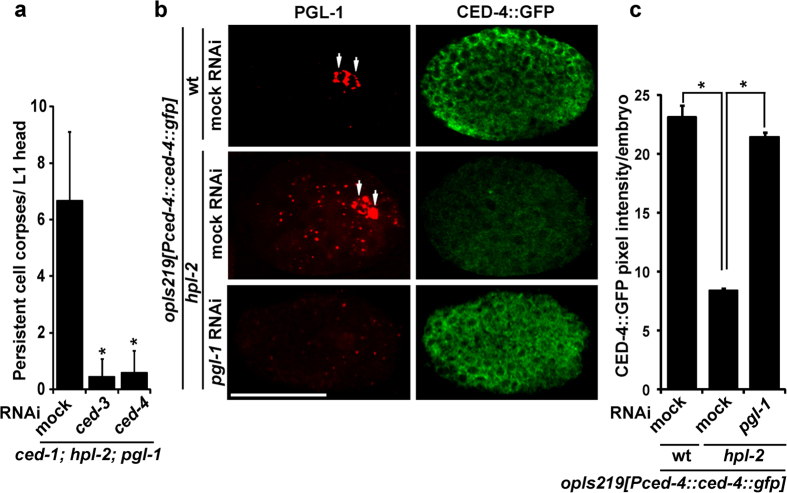
CED-4::GFP expression was reduced in *hpl-2*-mutant embryos due to ectopic expression of PGL-1. (**a**) Mean ± s.d. numbers of persistent cell corpses per L1 head at 25 °C (n > 50) in *ced-1(e1735); hpl-2(tm1489); pgl-1(ct131)* triple mutants with or without RNAi depletion of *ced-3* or *ced-4*. *P < 0.05 compared with mock RNAi control. (**b**) Expression of a *ced-4::gfp* transgene, *opIs219*, which is driven by its own *ced-4* promoter, in ca. 300-cell-stage embryos at 25 °C in wild type (wt) and *hpl-2(tm1489)* mutants with or without RNAi depletion of *pgl-1*. Expressions of PGL-1 (red) and CED-4::GFP (green) were detected by immunofluorescence staining with anti-PGL-1 (OIC1D4) and anti-GFP antibodies, respectively. Note the ectopic expression of PGL-1 in somatic blastomeres in the mock RNAi-treated *hpl-2* mutant embryo. Also note that PGL-1 signals were mostly eliminated in the *pgl-1* RNAi-treated *hpl-2* mutant embryo. Arrows: primordial germ cells, Z2 and Z3, which contained PGL-1-positive P granules. Scale bar: 20 μm. (**c**) Expression levels of CED-4::GFP in the *opIs219[Pced-4::ced-4::gfp]* transgenic embryos at 25 °C in wild-type (wt) and *hpl-2(tm1489)*-mutant backgrounds with or without RNAi depletion of *pgl-1,* which were digitally quantified from immunofluorescence images and shown as mean ± s.d. pixel intensities per embryo using an arbitrary unit. More than 50 embryos were quantified for each RNAi condition. *P < 0.05 compared with mock RNAi-treated *hpl-2(tm1489); opIs219[Pced-4::ced-4::gfp]* embryos.

To understand underlying mechanisms that contribute to a reduction in somatic apoptosis in *hpl-2* mutants, we examined whether expression level of any essential apoptosis regulator was affected in *hpl-2* mutants. In a previous study, we found that the protein level of CED-4, an adaptor protein similar to mammalian apoptotic protease-activating factor-1 (Apaf-1)^19^, increased in *pgl-1* mutant gonads, indicating that PGL-1 is a negative regulator of CED-4 expression in the germline^8^. We hypothesized that ectopic expression of PGL-1 in synMuv B-mutant somatic cells might repress CED-4 expression in the soma. First, we confirmed that somatic apoptosis in *ced-1; hpl-2; pgl-1* triple mutants was dependent on the activities of *ced-3* and *ced-4,* the core apoptotic machinery, because somatic apoptosis in the triple mutants was significantly suppressed by RNAi depletion of *ced-3* and *ced-4* (Fig. 2a). Next, we examined expression level of a *ced-4::gfp* transgene, *opIs219,* the transcription of which is controlled by its own *ced-4* promoter^20^, in ca. 300-cell-stage embryos in wild-type, *hpl-2* single-mutant, and *hpl-2; pgl-1(RNAi)* double-mutant backgrounds at 25 °C (Fig. 2b,c). We found that in *hpl-2*-mutant background, although PGL-1 was ectopically expressed in the somatic blastomeres, subcellular localizations of CED-4::GFP and PGL-1 were not overlapping. CED-4::GFP displayed a weblike pattern, suggesting that CED-4::GFP most likely localized to mitochondria, whereas PGL-1 displayed a granular pattern in the embryonic cells (Fig. 2b). There may exist an inverse correlation between localization of CED-4 and PGL-1 because when this *ced-4::gfp* transgene was expressed in wild-type background, the signal was substantially weaker in the primordial germ cells, Z2 and Z3, to which PGL-1 specifically localized, than in the neighboring somatic blastomeres in the transgenic embryos (Fig. S2a). Notably, CED-4::GFP overall expression was significantly reduced in *hpl-2*-mutant embryos compared to wild-type embryos (Fig. 2b,c). Reduction of CED-4::GFP expression compared to wild type was also observed in another synMuv B-class, *lin-13*-mutant embryos (Fig. S2b). This reduction of *ced-4* expression in *hpl-2* (and *lin-13*) mutants seems to occur at a post-transcriptional level, because mRNA levels of *ced-4* were not significantly different between wild-type N2 and *hpl-2* mutants at 25 °C (Fig. S2c; *p* = 0.83). Furthermore, CED-4::GFP expression was recovered to a higher level by RNAi depletion of *pgl-1* in *hpl-2*-mutant embryos compared to mock RNAi-treated *hpl-2*-mutant embryos (Fig. 2b,c). These results indicate that ectopic expression of PGL-1 in *hpl-2*-mutant somatic cells was responsible for the reduction in CED-4 expression in the soma, which likely contributes to repression of somatic apoptosis in *hpl-2* and other synMuv B-class mutants.

### Somatic apoptosis in *hpl-2* mutants increased following DNA damage in a *sir-2.1*-dependent manner

In germ cells, apoptosis increases following DNA damage compared to physiological conditions, which is termed DNA-damage-induced germline apoptosis^21^. By contrast, in wild-type somatic cells, the apoptosis level does not change before and after DNA damage^21^. It was shown that *sir-2.1*, which encodes a *C. elegans* Sirtuin homolog^22^, was essential for inducing a higher level of germline apoptosis following DNA damage^23^. During apoptosis, SIR-2.1 changes its subcellular localization from the nucleus to the cytoplasm and transiently colocalizes with CED-4, possibly to activate CED-3^23^. In a previous study, we identified that *sir-2.1* was essential for high levels of germline apoptosis in *pgl-1* and *pgl-3* mutants^8^. We also uncovered that cytoplasmic translocation of SIR-2.1 was substantially suppressed in wild-type germ cells by perinuclearly localized PGL-1 and PGL-3 to repress excessive germline apoptosis^8^. Therefore, we examined whether SIR-2.1 also plays a pivotal role in somatic apoptosis in synMuv B mutants.

**Figure 3:**
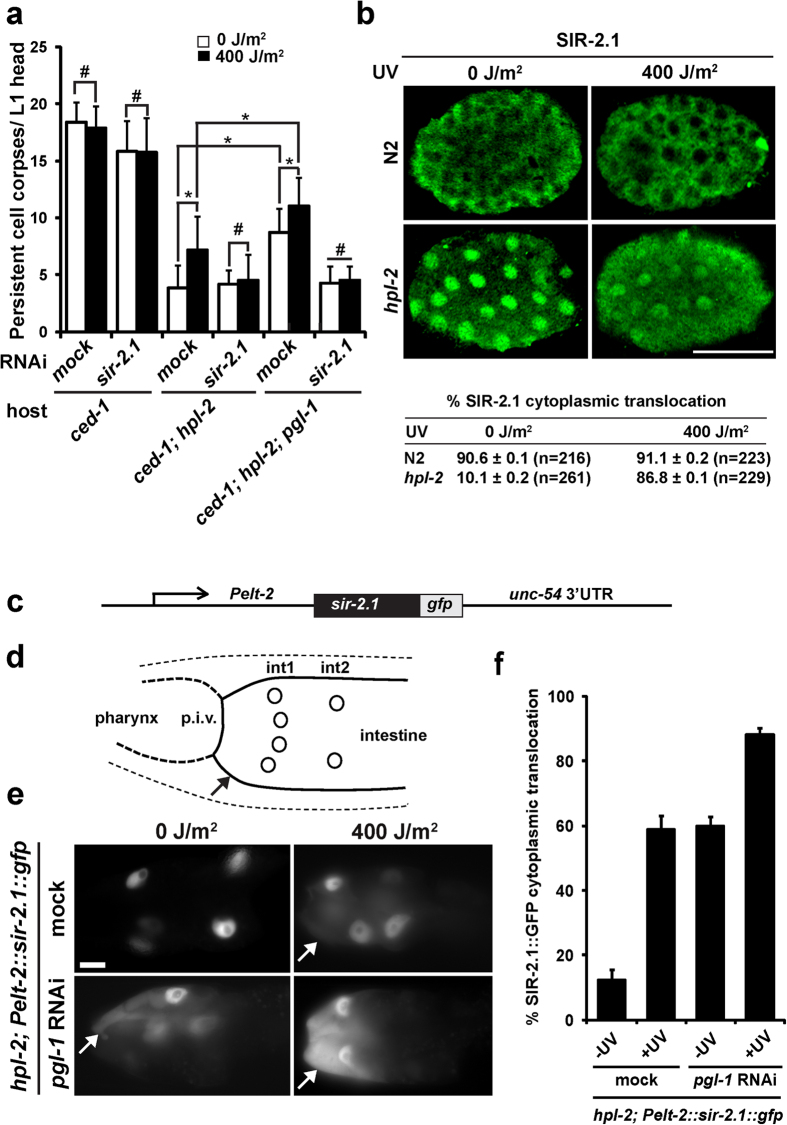
Somatic apoptosis in *hpl-2* mutants increased following UV irradiation in a *sir-2.1*-dependent manner. (**a**) Mean ± s.d. numbers of persistent cell corpses per L1 head at 25 °C (n > 50) in *ced-1(tm2420)* single, *ced-1(tm2420); hpl-2(tm1489)* double, and *ced-1(tm2420); hpl-2(tm1489); pgl-1(ct131)* triple mutants, which were treated or not treated with *sir-2.1* RNAi and irradiated (black bars) or not irradiated (white bars) with 400 J/m^2^ of UV. *P < 0.05. ^#^*P* > 0.05. (**b**) Subcellular localization of SIR-2.1 in N2 and *hpl-2* mutant embryos, which were irradiated (400 J/m^2^) or not irradiated (0 J/m^2^) with UV. Percent SIR-2.1 cytoplasmic translocation in each category of embryos is shown at the bottom. Scale bar: 20 μm. (**c**) Schematic diagram of a *sir-2.1* transgene introduced into *hpl-2(tm1489)* mutant animals, which is translationally fused with *gfp* and controlled by the *elt-2* promoter and *unc-54* 3′UTR. (**d**) Schematic diagram depicting anterior-end part of intestine in *C. elegans* adult hermaphrodite. The anterior end of the intestine consists of four int1 cells and two int2 cells^26^,^27^, and is connected to the pharynx via the pharyngeal-intestinal valve (p.i.v.). Circles: nuclei of int1 and int2 cells. Arrow: cytoplasm of intestinal cells. (**e**) Subcellular distribution of SIR-2.1::GFP expressed in intestinal cells under the control of the *elt-2* promoter in *hpl-2(tm1489); Ex[Pelt-2::sir-2.1::gfp]* transgenic animals at 25 °C, which were treated or not treated with *pgl-1* RNAi and irradiated or not irradiated with 400 J/m^2^ of UV. Arrows: cytoplasmically translocated SIR-2.1::GFP signals. Scale bar: 20 μm. (**f**) Mean ± s.d. percent of intestinal cells that showed cytoplasmic translocation of SIR-2.1::GFP in *hpl-2(tm1489); Ex[Pelt-2::sir-2.1::gfp]* transgenic animals at 25 °C with or without *pgl-1* RNAi depletion and with or without 400 J/m^2^ of UV irradiation.

We found that somatic apoptosis increased significantly following DNA damage generated by UV irradiation^24^, compared to non-irradiated conditions in *ced-1; hpl-2* double and *ced-1; hpl-2; pgl-1* triple mutants, but not in *ced-1* single, *ced-1; sir-2.1(RNAi)* double, *ced-1; hpl-2; sir-2.1(RNAi)* triple, and *ced-1; hpl-2; pgl-1 sir-2.1(RNAi)* quadruple mutants (Fig. 3a; compare pairs of white and black bars). Increase in somatic apoptosis following DNA damage was observed not only in *hpl-2* mutants but also in the other synMuv B mutants tested (Fig. S3a,b). These results strongly suggest that, in contrast to wild-type somatic apoptosis, somatic apoptosis in synMuv B mutants is DNA-damage inducible as is germline apoptosis. Furthermore, because the apoptosis levels failed to increase after DNA damage in *ced-1; hpl-2; sir-2.1(RNAi)* triple, *ced-1; hpl-2; pgl-1 sir-2.1(RNAi)* quadruple, and *ced-1; lin-13; sir-2.1(RNAi)* triple mutants (Fig. 3a and Fig. S3b), the increase in somatic apoptosis following DNA damage in synMuv B mutants is dependent on the activity of *sir-2.1,* as is DNA-damage-induced germline apoptosis. In addition, higher levels of somatic apoptosis in *ced-1; hpl-2; pgl-1* triple mutants than in *ced-1; hpl-2* double mutants under both physiological and DNA-damaged conditions (Fig. 3a) suggest that *pgl-1* is involved in suppression of *sir-2.1* activity during the course of somatic apoptosis in synMuv B mutants. From these results, we conclude that somatic apoptosis in synMuv B mutants, which is repressed under physiological conditions, increases following DNA damage in a *sir-2.1*-dependent manner, as does germline apoptosis.

In search for molecular basis that increases somatic apoptosis following DNA damage in synMuv B mutants but not in wild type, we UV-irradiated or not irradiated wild-type N2 and *hpl-2* mutant animals, and immunostained their embryos with anti-SIR-2.1 antibody (Fig. 3b). We found that even without UV irradiation, SIR-2.1 was more significantly translocated to the cytoplasm in N2 embryos (90.6 ± 0.1%, n = 216) than in *hpl-2* mutant embryos (10.1 ± 0.2%, n = 261), when they developed at 25 °C (Fig. 3b). With UV irradiation, the frequency of SIR-2.1 cytoplasmic translocation significantly increased in *hpl-2* mutant embryos (86.8 ± 0.1%, n = 229), while this frequency stayed high in N2 embryos (91.1 ± 0.2%, n = 223) (Fig. 3b). These observations suggest that somatic apoptosis in *hpl-2* mutants was maintained at a low level under physiological conditions (Fig. 3a), at least partly by substantial nuclear retention of SIR-2.1 (Fig. 3b). The level of somatic apoptosis in *ced-1; hpl-2* double mutants was increased to a higher level following DNA damage (Fig. 3a), due to significant cytoplasmic translocation of SIR-2.1 (Fig. 3b). By contrast, in wild type (*ced-1* single mutants), the level of somatic apoptosis was already high under physiological conditions (Fig. 3a), due to significant cytoplasmic translocation of SIR-2.1 without DNA damage (Fig. 3b). The level of somatic apoptosis did not change (stayed high) following DNA damage in wild type (*ced-1* single mutants) (Fig. 3a), because the frequency of SIR-2.1 cytoplasmic translocation stayed high following DNA damage (Fig. 3b). We consider that in wild-type (non-synMuv B-mutant) somatic cells, SIR-2.1 is not a critical factor that affects the level of developmental apoptosis, because the majority of SIR-2.1 is consistently translocated to the cytoplasm with or without DNA damage as observed (Fig. 3b). Currently, it is not clear why the levels of somatic apoptosis were higher in *ced-1; sir-2.1(RNAi)* double mutants than in *ced-1; hpl-2; sir-2.1(RNAi)* triple or *ced-1; hpl-2; pgl-1 sir-2.1(RNAi)* quadruple mutants, although *sir-2.1* was depleted in all the three mutants (Fig. 3a). Indeed, the levels of somatic apoptosis in *ced-1; sir-2.1(RNAi)* double mutants were not significantly different from those in *ced-1* single mutants, as previously reported (Fig. 3a)^23^. One possible hypothesis is that although SIR-2.1 is one of the most critical factors that determine the levels of apoptosis in synMuv B-mutant backgrounds, other factors such as CED-4, which was shown to be more abundantly expressed in wild type than in synMuv B mutants (Fig. 2b,c and Fig. S2b), can be more crucial than SIR-2.1 in non-synMuv B-mutant backgrounds to determine the levels of apoptosis. In conclusion, the above results strongly suggest that SIR-2.1 plays a critical role in the control of somatic apoptosis in synMuv B mutants but not in wild type. These results also suggest that somatic apoptosis in synMuv B mutants is controlled by a mechanism that mimics DNA-damage-induced germline apoptosis, in which PGL-1 and SIR-2.1 play critical roles.

### Cytoplasmic translocation of SIR-2.1 was substantially suppressed by ectopically expressed PGL-1 in *hpl-2*-mutant intestinal cells

To further investigate underlying mechanisms, we expressed a *sir-2.1::gfp* transgene in *hpl-2*-mutant intestinal cells under the control of the *elt-2* promoter (Fig. 3c). *elt-2* is specifically expressed in the intestine throughout the animal’s life^25^. We expressed SIR-2.1::GFP in the intestine because intestinal cells are large somatic cells^26^,^27^; hence, differences in SIR-2.1::GFP subcellular localization are readily distinguishable (Fig. 3d). In addition, in *hpl-2*-mutant intestinal cells, PGL-1 and PGL-3 were often observed as perinuclear granules, as are P granules in germ cells, at elevated temperatures^9^. We found that cytoplasmic translocation of SIR-2.1::GFP occurred only in a small proportion of *hpl-2*-mutant intestinal cells without UV irradiation (Fig. 3e,f; 12.2 ± 3.4%, n = 278). However, after UV irradiation, which was previously shown to cause elimination of PGL-1 and PGL-3 from germ cells^8^, the proportion of intestinal cells that showed SIR-2.1::GFP cytoplasmic translocation substantially increased (Fig. 3e,f; 58.9 ± 4.1%, n = 258). Further, when *pgl-1* was depleted by RNAi in *hpl-2* mutants, a substantial proportion of intestinal cells showed SIR-2.1::GFP cytoplasmic translocation even without UV irradiation (Fig. 3e,f; 59.7 ± 3.1%, n = 226). Moreover, after UV irradiation, cytoplasmic translocation of SIR-2.1::GFP occurred in the majority of intestinal cells in *pgl-1* RNAi-treated *hpl-2* mutants (Fig. 3e,f; 88.2 ± 2.0%, n = 407). These results indicate that cytoplasmic translocation of SIR-2.1 was substantially suppressed by ectopically expressed PGL-1 in *hpl-2*-mutant intestinal cells. We assume that this suppression also occurs in somatic cells that are programmed to die in *hpl-2* mutants. We consider that suppression of SIR-2.1 cytoplasmic translocation by ectopically expressed PGL-1 is one of the key mechanisms for repression of somatic apoptosis in *hpl-2* mutants. Therefore, failure of this suppression in *ced-1; hpl-2; pgl-1* triple mutants or by UV irradiation leads to increased somatic apoptosis compared to *ced-1; hpl-2* double mutants under physiological conditions (Fig. 3a).

### Somatic apoptosis was reduced by ectopic expression of *pgl-1* and *pgl-3* transgenes in wild-type somatic cells

**Figure 4:**
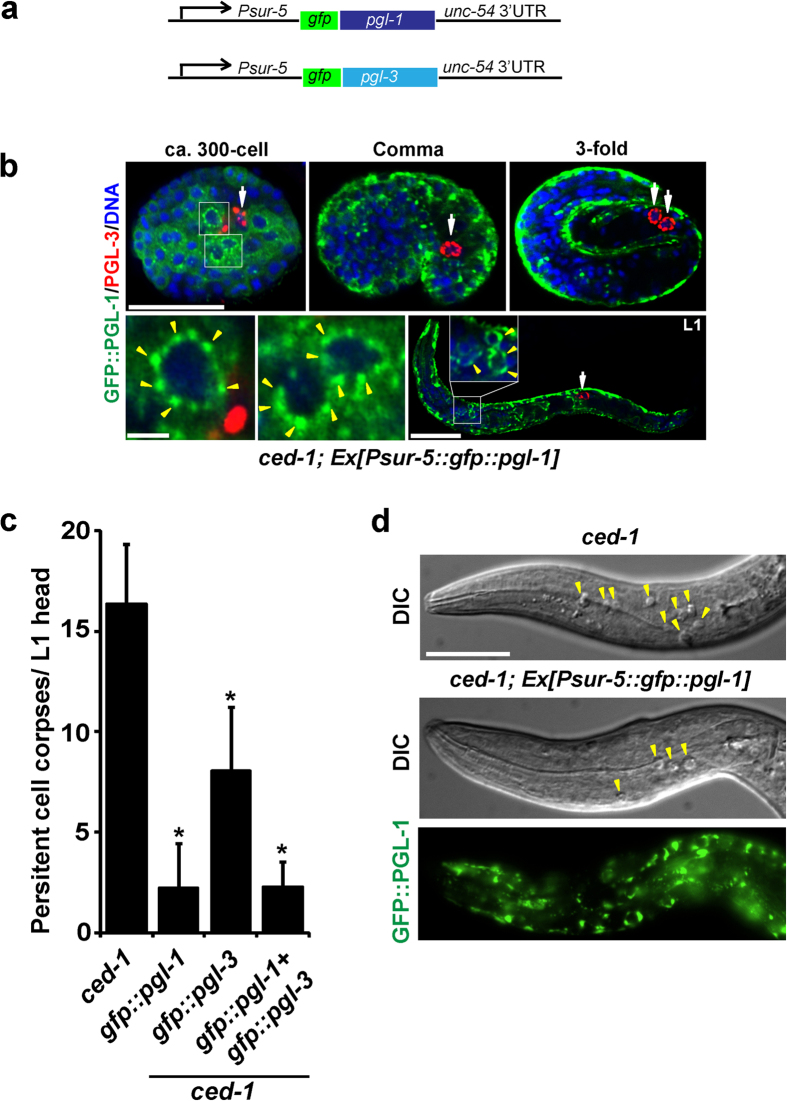
Somatic apoptosis in *ced-1* mutants was reduced by ectopic expression of *pgl-1* and *pgl-3* transgenes in the soma. (**a**) Schematic diagram of *pgl-1* and *pgl-3* transgenes introduced into *ced-1(tm2420)* mutant animals, which are translationally fused with *gfp* and controlled by the *sur-5* promoter and *unc-54* 3′UTR. (**b**) The *sur-5* promoter-driven *gfp::pgl-1* transgene was expressed in most somatic cells throughout development in transgenic animals. The transgenic animal’s embryos at around the 300-cell stage, comma stage, 3-fold stage, and an L1 larva, were co-immunostained with anti-GFP (green) and anti-PGL-3 (red) antibodies together with TO-PRO-3 DNA staining (blue). Scale bars in whole images: 20 μm. Scale bar in enlarged images: 2 μm. Yellow arrowheads in the enlarged images indicate some of GFP::PGL-1-positive granules localized to the perinuclear region of unidentified somatic cells. White arrows indicate primordial germ cells, Z2 and Z3, which contained PGL-3-positive perinuclear P granules. (**c**) Mean ± s.d. numbers of persistent cell corpses per L1 head (n > 50) in *ced-1(tm2420)* mutants and *ced-1(tm2420)* mutants that contained a transgene *Ex[Psur-5::gfp::pgl-1], Ex[Psur-5::gfp::pgl-3],* or *Ex[Psur-5::gfp::pgl-1 + Psur-5::gfp::pgl-3],* which were grown at 20 °C. *P < 0.05. (**d**) L1 larval heads of *ced-1(tm2420)* mutant animal and *ced-1(tm2420)* mutant animal that contained the *Ex[Psur-5::gfp::pgl-1]* transgene displayed different numbers of persistent cell corpses at 20 °C. Yellow arrowheads in DIC images indicate some of the cell corpses. Fluorescence signal of GFP::PGL-1 in the head part of the transgenic animal is also shown. Scale bar: 20 μm.

Finally, we asked whether ectopically expressed PGL-1 and PGL-3 repress somatic apoptosis only in a synMuv B-mutant background, in which various germline-specific proteins are co-expressed in addition to PGL-1 and PGL-3^9^, or also in a wild-type background, in which no other germline-specific proteins are co-expressed. To assess this, we ectopically expressed *gfp::pgl-1* and *gfp::pgl-3* transgenes in *ced-1*-mutant somatic cells under the control of the *sur-5* promoter (Fig. 4a). *sur-5* is strongly expressed in most of the somatic cells throughout *C. elegans* development^28^. We found that GFP::PGL-1 and GFP::PGL-3 were ectopically expressed in most of the somatic cells in transgenic animals throughout development, including the embryonic stage (Fig. 4b), during which the majority of programmed cell death takes place^29^. We also observed that, in some unidentified somatic cells in embryos and larvae, GFP::PGL-1 and GFP::PGL-3 were present as perinuclear granules, as were P granules in Z2 and Z3 primordial germ cells (Fig. 4b; yellow arrowheads vs. white arrows). Intriguingly, we found that somatic apoptosis levels were significantly reduced in the transgenic animals compared to the control *ced-1*-mutant animals even at a permissive temperature, 20 °C (Fig. 4c,d). We consider that observed self-association of GFP::PGL-1 and GFP::PGL-3 into perinuclear granules in somatic cells in the transgenic animals possibly bypassed the requirement of other germline proteins, such as GLH-1 and GLH-4, for suppression of apoptosis in the soma. It was previously shown that GLH-1 and GLH-4 were required for localization of PGL-1 and PGL-3 to P granules in the germline^5^,^8^. On the other hand, an ability of PGL-1 and PGL-3 to self-associate into granules in the absence of other germline proteins was demonstrated using a cultured mammalian cell system^30^. Therefore, if GFP::PGL-1 or GFP::PGL-3 can self-associate into perinuclear granules in somatic cells as observed (Fig. 4b), the functions of GLH-1 and GLH-4 are no longer necessary and these germline proteins would become dispensable for suppressing apoptosis in the soma. In addition, we found that ectopically expressed GFP::PGL-1 substantially suppressed cytoplasmic translocation of SIR-2.1 in the transgenic embryos as in *hpl-2* mutant embryos (Fig. 3b and Fig. S4). Although in the absence of *pgl-1* RNAi (mock), only 29% (n = 27) of the transgenic embryos showed SIR-2.1 cytoplasmic translocation, after *pgl-1* RNAi depletion, as much as 88% (n = 26) of the transgenic embryos showed substantial SIR-2.1 cytoplasmic translocation (Fig. S4). These results suggest that overlapping molecular mechanisms operate between the ectopic *pgl-1* (or *pgl-3*) transgenic animals and synMuv B mutants for the control of somatic apoptosis. These results indicate that somatic ectopic expression of PGL-1 and/or PGL-3 represses somatic apoptosis not only in synMuv B mutants but also in a wild-type background. Therefore, we conclude that PGL-1 and PGL-3 are both necessary and sufficient to repress apoptosis in *C. elegans* cells.

In a previous study, it was reported that long-lived *C. elegans daf-2* mutants ectopically expressed several germline-specific genes including *pgl-1* and *pgl-3* in their somatic cells^31^. Intriguingly, in that study, RNAi depletion of *pgl-1* and *pgl-3* resulted in a decrease in lifespan in *daf-2* mutants, suggesting that somatic ectopic expression of PGL-1 and PGL-3 contributed to the lifespan extension in *daf-2* mutants^31^. However, in a recent follow-up study, Knutson *et al*.^32^ found no evidence of P-granule protein expression in *daf-2* mutant somatic cells, or of extended longevity in synMuv B mutants^32^. Therefore, currently it is controversial whether somatic expression of PGL-1 or PGL-3 really contributes to lifespan extension of *C. elegans* individuals. In this study, we demonstrated that although PGL-1 and PGL-3 are originally designed to serve as germline-specific apoptosis repressors^8^, they could also function as negative regulators of programmed cell death when introduced into the soma. This result raises a fascinating possibility that expression of a germline program in somatic cells can provide the soma with partial germline immortality at a cellular level. If this is the case, our finding may provide a novel clue to develop a treatment for diseases that arise from excess cell death such as Parkinson’s or Alzheimer’s diseases^33^,^34^.

## Methods

### *C. elegans* strains and maintenance

All strains were maintained at either 15 or 20 °C on Nematode Growth Medium (NGM) agar plates seeded with *Escherichia coli* OP50, according to standard protocols^35^. When synMuv B-mutant phenotype was examined, gravid adult hermaphrodites were shifted from 20 °C to 25 °C, and their progeny developed at 25 °C were analyzed for their phenotype. The following mutant alleles were used. LG I: *unc-13(e51)*, *ced-1(e1735)*, *ced-1(tm2420)*; LG III: *hpl-2(tm1489)*, *lin-13(ok838)*; LG IV: *pgl-1(ct131)*, *him-3(e1147)*, *opIs219[Pced-4::ced-4::gfp]*. The following extrachromosomal arrays were used. *kkuEx10[Psur-5::gfp::pgl-1* + *rol-6(su1006)]*, *kkuEx11[Psur-5::gfp::pgl-3* + *rol-6(su1006)]*, *kkuEx12[Psur-5::gfp::pgl-1* + *Psur-5::gfp::pgl-3* + *rol-6(su1006)]*, *kkuEx13[Pelt-2::sir-2.1::gfp* + *rol-6(su1006)]*. Cell corpses were counted in *ced-1(e1735, tm2420)* mutant background. Therefore, either *ced-1(e1735)* single or *ced-1(tm2420)* single mutant was used as a wild-type control for the analysis. For the other analyses, the strain N2 was used as a wild-type control. Some strains were obtained from the Caenorhabditis Genetics Center (CGC) or from National BioResource Project (NBRP).

### Construction of transgenic animals

To construct the *Pelt-2::sir-2.1::gfp::unc-54 3*′*UTR* vector, pMAA1, first, *sir-2.1* genomic sequence was fused with *gfp* sequence by fusion PCR^36^. Second, *mCherry* sequence in pOLB1872 vector (a gift from Olaf Bossinger), which was flanked by *elt-2* promoter and *unc-54* 3′UTR sequences in the vector, was replaced with this *sir-2.1::gfp* fused sequence using In-Fusion HD cloning kit (Clontech) to construct pMAA1. pMAA1 was then co-microinjected with pRF4, which expresses dominant roller gene, *rol-6(su1006)*, into N2 adult hermaphrodite gonads to establish a heritable Roller transgenic line expressing *Pelt-2::sir-2.1::gfp*, as previously described^37^. Then, this transgenic line was crossed with *hpl-2(tm1489)* mutants to construct the transgenic line, YHS140: *hpl-2(tm1489) III; kkuEx13[Pelt-2::sir-2.1::gfp* + *rol-6(su1006)]*. To construct the *Psur-5::gfp::pgl-1::unc-54 3*′*UTR* vector, pMAA2, and the *Psur-5::gfp::pgl-3::unc-54 3*′*UTR* vector, pMAA3, first, luciferase sequence was removed from the plasmid, pSLGCV (Addgene, No. 49862), which contains *sur-5* promoter, *gfp* sequence, and *unc-54 3*′*UTR*^38^. Then, *pgl-1* and *pgl-3* genomic sequences were inserted into the junction between *gfp* sequence and *unc-54 3*′*UTR* of pSLGCV using In-Fusion HD cloning kit (Clontech) to construct pMAA2 and pMAA3, respectively. Then, pMAA2 and/or pMAA3 were co-microinjected with pRF4 into *ced-1(tm2420)* mutant adult hermaphrodite gonads to establish the following heritable Roller transgenic lines. YHS133: *ced-1(tm2420) I; kkuEx10[Psur-5::gfp::pgl-1* + *rol-6(su1006)]*. YHS134: *ced-1(tm2420) I; kkuEx11[Psur-5::gfp::pgl-3* + *rol-6(su1006)].* YHS135: *ced-1(tm2420) I; kkuEx12[Psur-5::gfp::pgl-1* + *Psur-5::gfp::pgl-3* + *rol-6(su1006)].*

### Cell corpse counting

Numbers of persistent cell corpses in the head region of newly-hatched L1 larvae were scored as previously described^13^,^14^. Briefly, synchronized L1 larvae, which were newly hatched in the absence of food, were anesthetized in M9 buffer containing 0.2 mM tetramisole (Sigma), mounted on a 2% agarose pad on a glass slide, covered with a coverslip, and sealed with vaseline. Then, the numbers of persistent cell corpses in the head region were counted under Nomarski DIC microscopy. Apoptotic cells have a characteristic button-like morphology and are easily distinguishable from living cells^29^. Throughout this study, we counted the number of cell corpses under *ced-1* mutant background, because in *ced-1* mutants, engulfment of cell corpses is blocked, and therefore, a more sensitive condition to count cell corpses is provided than N2^13^,^14^. When the numbers of cell corpses were counted in animals that contained a synMuv B mutation, their mothers were shifted from 20 °C to 25 °C, and their L1 progeny grown at 25 °C through the development were scored for their cell corpse numbers.

### UV irradiation

L4 mother hermaphrodites were pre-cultured at 20 °C for 24 h, irradiated or not irradiated with 400 J/m^2^ of UV-C light (254 nm) on OP50-seeded NGM plates, post-cultured on the same plates at 25 °C for 16 h, then transferred to a drop of M9 buffer on a printed glass-slide well. The mother hermaphrodites in M9 buffer were further incubated at 25 °C for 24 h in a wet chamber, and finally their laid progeny, which were grown at 25 °C and synchronized at the L1 larval stage in the absence of food, were examined for the number of persistent cell corpses in their head region.

### RNAi

RNAi analysis was performed using the “RNAi-by-soaking” method as previously described^39^. dsRNA for *lin-9, lin-35, lin-37, lin-54, pgl-1, pgl-3, glh-1, glh-4, cgh-1, car-1, ced-3, ced-4,* and *sir-2.1* genes was synthesized by T7 RNA polymerase-mediated *in vitro* transcription from respective cDNA templates flanked by T7 promoter sequences, which were PCR-amplified from respective yk cDNA clones in lambda ZAPII vectors (gifts from Yuji Kohara) using T7 primer (5′-GTAATACGACTCACTATAGGGC-3′) and CMo422 primer (5′-GCGTAATACGACTCACTATAGGGAACAAAAGCTGGAGCT-3′). L4 larvae of appropriate genotype were soaked with each dsRNA solution for 24 h, recovered to OP50-seeded NGM plates, irradiated with UV when required, further incubated on the plates at 25 °C for 24 h, and resulting RNAi phenotypes of themselves or their progeny were analyzed.

### Immunofluorescence

Embryos obtained from dissected gravid adult hermaphrodites or synchronized L1-stage larvae were placed on poly-lysine-coated glass slides, freeze-cracked with liquid nitrogen, fixed with cold methanol and cold acetone, and immunostained with primary and secondary antibodies as previously described^5^,^6^,^8^. The following primary and secondary antibodies were used: rabbit anti-PGL-1 antibody^5^ (1:4,000), rabbit anti-GFP antibody (1:400; Molecular Probes), mouse monoclonal OIC1D4 antibody that specifically recognizes PGL-1 (undiluted; Developmental Studies Hybridoma Bank), rat anti-PGL-3 antibody^6^ (1:1,000), rabbit anti-SIR-2.1 antibody (1:500; Novus), Alexa Fluor 488 goat anti-rabbit IgG (1:500; Molecular Probes), Alexa Fluor 546 goat anti-mouse IgG (1:500; Molecular Probes), and Alexa Fluor 546 goat anti-rat IgG (1:500; Molecular Probes). The specimens were further counter stained with 1 μM TO-PRO-3 (Molecular Probes) to stain DNA. Immunofluorescence images were acquired using a confocal microscope (Olympus, FV1000 Spectral).

### Image analysis

Specimens on glass slides were observed under a fluorescence microscope (Zeiss, Axioskop 2 MOT), and their images were acquired using a cooled CCD digital camera (Hamamatsu, ORCA-ER) with operation software (Nikon, NIS-Elements). Images were acquired from multiple embryos or animals under the same exposure conditions, and mean pixel intensities of fluorescence images were calculated using Image J software (http://rsb.info).

### qRT-PCR

Wild-type N2 and *hpl-2(tm1489)* mutant mothers were shifted from 20 °C to 25 °C, and their progeny were grown on OP50-seeded NGM agar plates until the adult stage at 25 °C. Then, ca. 400 each of those gravid adult hermaphrodite progeny were harvested for RNA extraction. Total RNA was extracted with Trizol reagent (Sigma), purified, and reverse transcribed with M-MLV reverse transcriptase (Gibco BRL, USA) using an oligo-dT primer (Promega, USA) to synthesize the first-strand cDNA. *ced-4* cDNA product was PCR-amplified using *ced-4* (C35D10.9) F primer; 5′-AAA CTA TCG CCA ATG GAA TCT C-3′, and *ced-4* (C35D10.9) R primer; 5′-TTT GGA TAC ATC TCA CTG GCT-3′. *act-1* cDNA product, which was used as an internal control, was PCR-amplified using *act-1* F primer; 5′-CCAGGAATTGCTGATCGTATGCAGAA-3′, and *act-1* R primer; 5′-TGGAGAGGGAAGCGAGGATAGA-3′. PCR reactions were performed in a 25 μl reaction volume using Power SYBR Green PCR Master Mix (Applied Biosystems, USA). mRNA levels of *ced-4* in N2 and *hpl-2* mutants were quantified by averaging triplicate qRT-PCR measurements, and normalized against that of *act-1* for comparison.

### Data presentation and statistical analysis

All experiments were repeated more than three times for statistical analysis. Data in graphs were presented as mean ± s.d. Statistical comparison of groups was carried out using Student’s *t*-test. We tested at least 50 samples for each independent experiment. Differences were considered as significant when *P* < 0.05.

## Additional Information

**How to cite this article**: Al-Amin, M. *et al*. Somatically expressed germ-granule components, PGL-1 and PGL-3, repress programmed cell death in *C. elegans*. *Sci. Rep.* **6**, 33884; doi: 10.1038/srep33884 (2016).

## Supplementary Material

Supplementary Information
