# Supplementary material for: Somatically expressed germ-granule components, PGL-1 and PGL-3, repress programmed cell death in C. elegans
Source: Sci Rep. 2016 Sep 21;6:33884. doi: 10.1038/srep33884 (PMC5030653; doi:10.1038/srep33884)
Supplement: Supplementary Information [file srep33884-s1.pdf]

Somatically expressed germ-granule components, PGL-1 and PGL-3, repress programmed cell death in *C. elegans*

Mohammad Al-Amin, Hyemin Min, Yhong-Hee Shim & Ichiro Kawasaki

Department of Bioscience and Biotechnology, Konkuk University, Seoul, Republic of Korea

Correspondence should be addressed to I. K. (ikawasak@mac.com)

or Y. H. S. (yshim@konkuk.ac.kr).

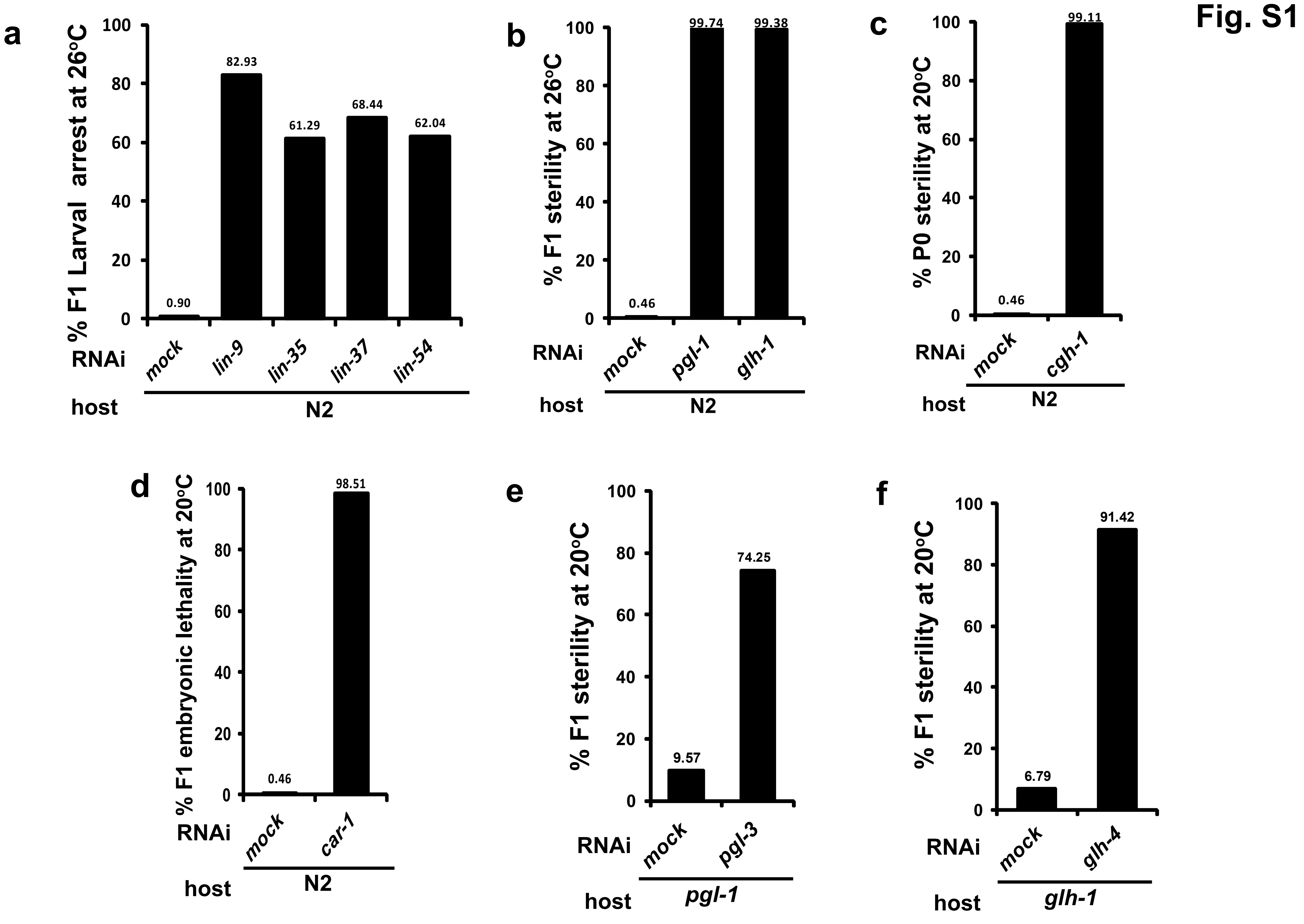
Fig. S1
a
% F1 Larval arrest at 26°C
0.90
82.93
61.29
68.44
62.04
RNAi
mock
lin-9
lin-35
lin-37
lin-54
host
N2
b
% F1 sterility at 26°C
0.46
99.74
99.38
RNAi
mock
pgl-1
glh-1
host
N2
c
% P0 sterility at 20°C
0.46
99.11
RNAi
mock
cgh-1
host
N2
d
% F1 embryonic lethality at 20°C
0.46
98.51
RNAi
mock
car-1
host
N2
e
% F1 sterility at 20°C
9.57
74.25
RNAi
mock
pgl-3
host
pgl-1
f
% F1 sterility at 20°C
6.79
91.42
RNAi
mock
glh-4
host
glh-1

Figure S1. Our RNAi treatments successfully phenocopied representative loss-of-function mutant phenotypes of target genes. (**a**) RNAi depletion of synMuv B-class genes, *lin-9*, *lin-35*, *lin-37*, and *lin-54,* in wild-type N2 L4 hermaphrodites caused high level of larval developmental arrest in their progeny at 26°C. (**b**) RNAi depletion of P-granule component genes, *pgl-1* and *glh-1,* in N2 L4 hermaphrodites caused high level of sterility in their progeny at 26°C. (**c**) RNAi depletion of a P-granule component gene, *cgh-1*, in N2 L4 hermaphrodites made them highly sterile at 20°C. (**d**) RNAi depletion of a P-granule component gene, *car-1*, in N2 L4 hermaphrodites caused high level of embryonic lethality in their progeny at 20°C. (**e**) RNAi depletion of a P-granule component gene, *pgl-3*, in *pgl-1* mutant L4 hermaphrodites caused high level of sterility in their progeny at 20°C. (**f**) RNAi depletion of a P-granule component gene, *glh-4*, in *glh-1* mutant L4 hermaphrodites caused high level of sterility in their progeny at 20°C.

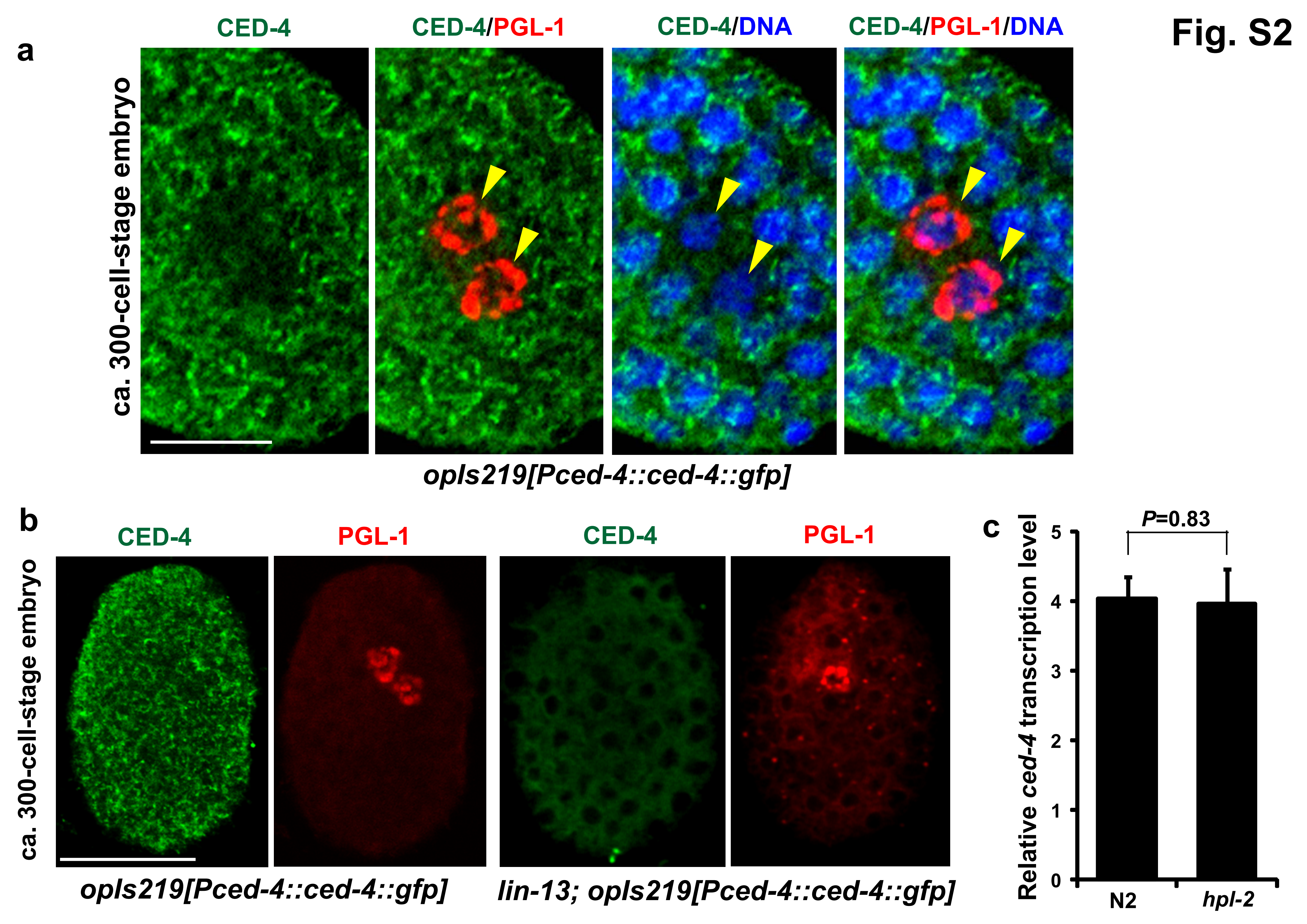
Fig. S2
a
CED-4
CED-4/PGL-1
CED-4/DNA
CED-4/PGL-1/DNA
ca. 300-cell-stage embryo
opIs219[Pced-4::ced-4::gfp]
b
CED-4
PGL-1
CED-4
PGL-1
ca. 300-cell-stage embryo
opIs219[Pced-4::ced-4::gfp]
lin-13; opIs219[Pced-4::ced-4::gfp]
c
P=0.83
Relative ced-4 transcription level
0
1
2
3
4
5
N2
hpl-2

Figure S2. CED-4::GFP expression was inversely correlated with PGL-1 expression. (**a**) Expression of CED-4::GFP (green) in a ca. 300-cell-stage *opIs219[Pced-4::ced-4::gfp]* transgenic embryo. PGL-1 (red) and DNA (blue) signals are also shown. Arrowheads (yellow) indicate primordial germ cells, Z2 and Z3, to which PGL-1 was specifically localized. Note that CED-4::GFP signal was substantially weaker in the Z2 and Z3 primordial germ cells than in the neighboring somatic blastomeres in the embryo. Scale bar: 10 μm. (**b**) Expression of CED-4::GFP (green) and PGL-1 (red) in *opIs219[Pced-4::ced-4::gfp]* transgenic embryos under either wild-type (left) or *lin-13* mutant (right) background at 25°C. Besides PGL-1 ectopic expression in the somatic blastomeres, CED-4::GFP overall expression was significantly reduced in *lin-13*-mutant background compared to wild-type background. Scale bar: 20 μm. (**c**) Relative mRNA expression level of *ced-4* in wild-type N2 and *hpl-2* mutants at 25ºC, measured by qRT-PCR. mRNA levels of *ced-4* were not significantly different between N2 and *hpl-2* mutants (*P*=0.83).

Fig. S3

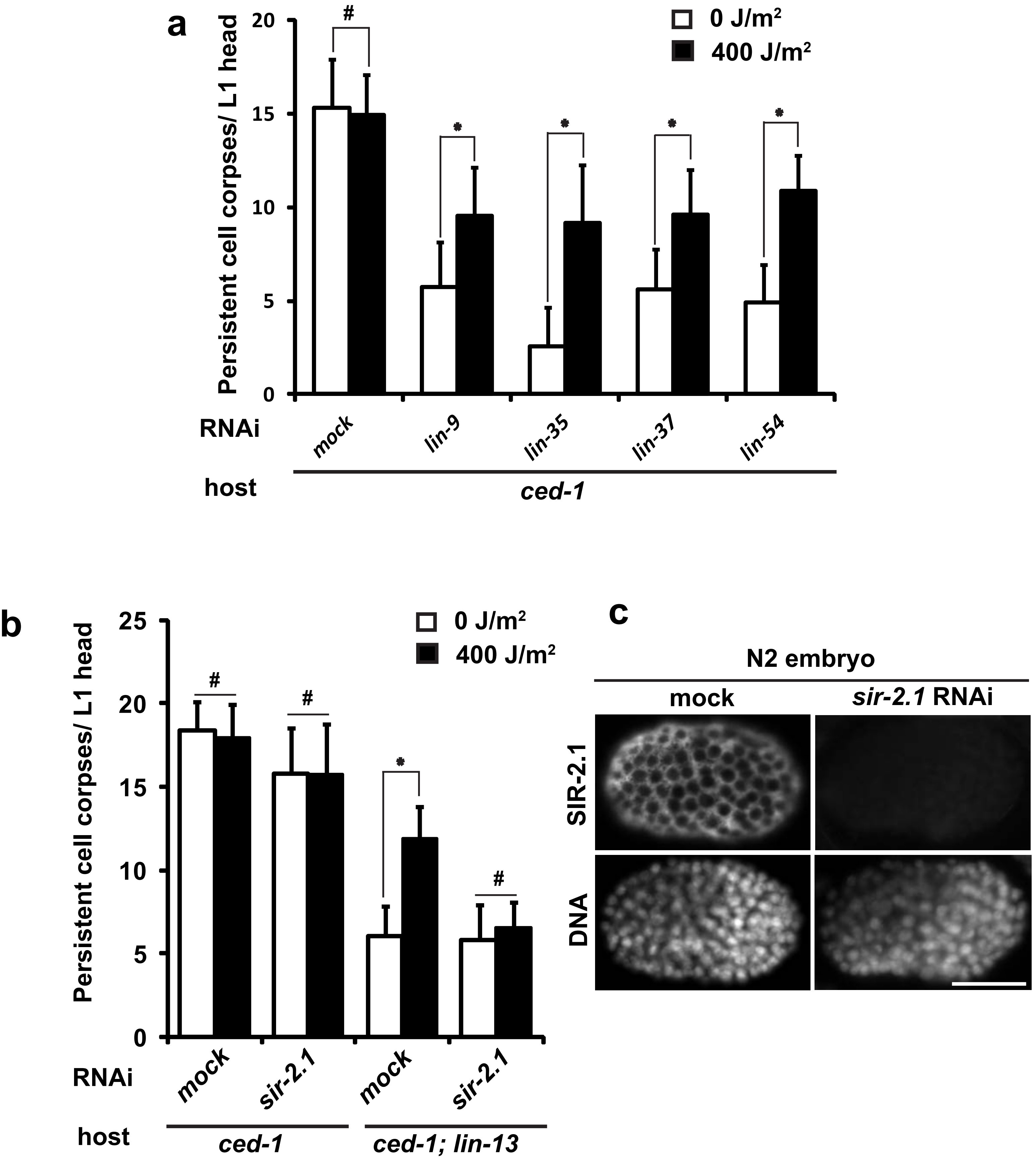
a
0 J/m²
400 J/m²
Persistent cell corpses/ L1 head
20
15
10
5
0
#
*
RNAi
mock
lin-9
lin-35
lin-37
lin-54
host
ced-1
b
0 J/m²
400 J/m²
Persistent cell corpses/ L1 head
25
20
15
10
5
0
RNAi
mock
sir-2.1
host
ced-1
ced-1; lin-13
c
N2 embryo
mock
sir-2.1 RNAi
SIR-2.1
DNA

Figure S3. Somatic apoptosis in synMuv B mutants increased following DNA damage in a *sir-2.1*-dependent manner. (**a**) Mean ± s.d. numbers of persistent cell corpses per L1 head at 25°C (n > 50) in *ced-1(tm2420)* mutants after RNAi depletion of *lin-9*, *lin-35*, *lin-37*, and *lin-54* gene, respectively, with mock RNAi control, which were irradiated (black bars) or not irradiated (white bars) with 400 J/m^2 of UV. $^{*}P < 0.05$. $^{\#}P > 0.05$. (**b**) Mean ± s.d. numbers of persistent cell corpses per L1 head at 25°C (n > 50) in *ced-1(tm2420)* single and *ced-1(tm2420); lin-13(ok838)* double mutants, which were treated or not treated with *sir-2.1* RNAi and irradiated (black bars) or not irradiated (white bars) with 400 J/m^2 of UV. $^{*}P < 0.05$. $^{\#}P > 0.05$. (**c**) SIR-2.1 expression in ca. 300-stage-cell embryos, which were treated or not treated with *sir-2.1* RNAi. SIR-2.1 signal was significantly depleted by *sir-2.1* RNAi treatment. Scale bar: 20 μm.

Fig. S4

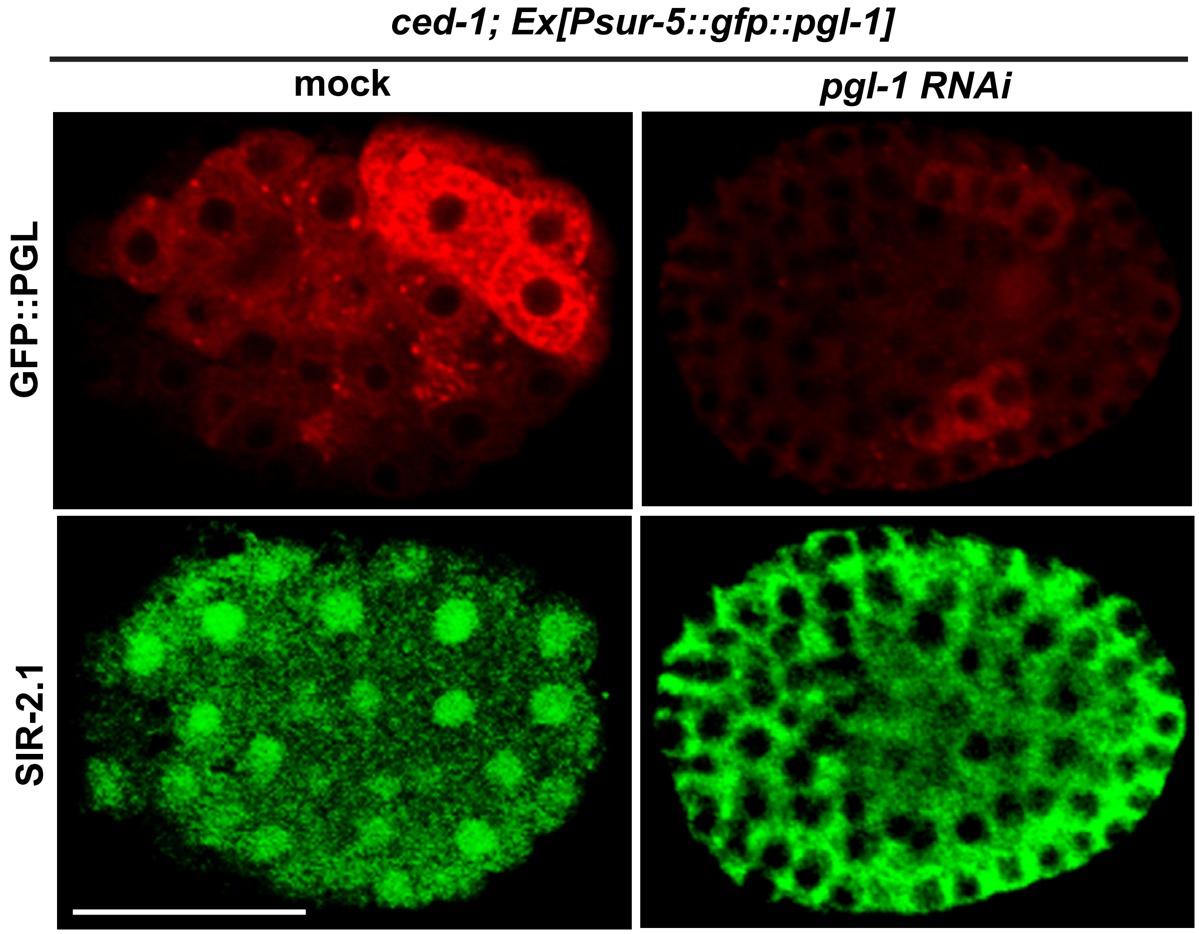

Figure S4. Ectopic expression of GFP::PGL-1 in somatic cells substantially suppressed cytoplasmic translocation of SIR-2.1 in *ced-1; Ex[Psur-5::gfp::pgl-1]* transgenic embryos. In the absence of *pgl-1 RNAi* (mock), only 29% (n=27) of the transgenic embryos showed SIR-2.1 (green) cytoplasmic translocation due to GFP::PGL-1 (red) somatic expression. After *pgl-1 RNAi* depletion, as much as 88%(n=26) of the transgenic embryos showed substantial SIR-2.1 cytoplasmic translocation due to loss of GFP::PGL-1. Expression of GFP::PGL-1 and SIR-2.1 was detected by immunofluorescence staining with anti-GFP and anti-SIR-2.1 antibody, respectively. Scale bar: 20 μm.
